# Genome-Wide Bioinformatics Analysis of *MAPK* Gene Family in Kiwifruit (*Actinidia Chinensis*)

**DOI:** 10.3390/ijms19092510

**Published:** 2018-08-24

**Authors:** Gang Wang, Tao Wang, Zhan-Hui Jia, Ji-Ping Xuan, De-Lin Pan, Zhong-Ren Guo, Ji-Yu Zhang

**Affiliations:** Institute of Botany, Jiangsu Province and Chinese Academy of Sciences, Nanjing 210014, China; wg20092011@163.com (G.W.); immmorer@163.com (T.W.); 13915954315@163.com (Z.-H.J.); xuanjiping@cnbg.net (J.-P.X.); PPxsperfect@163.com (D.-L.P.); zhongrenguo@cnbg.net (Z.-R.G.)

**Keywords:** mitogen-activated protein kinase (MAPK), kiwifruit, phylogenetic relationships, gene expression, biotic and abiotic stresses

## Abstract

Mitogen activated protein kinase (MAPK) cascades are universal signal transduction modules that play crucial roles in various biotic and abiotic stresses, hormones, cell division, and developmental processes in plants. Mitogen activated protein kinase (MAPK/MPK), being a part of this cascade, performs an important function for further appropriate cellular responses. Although MAPKs have been investigated in several model plants, no systematic analysis has been conducted in kiwifruit (*Actinidia chinensis*). In the present study, we identified 18 putative MAPKs in the kiwifruit genome. This gene family was analyzed bioinformatically in terms of their chromosome locations, sequence alignment, gene structures, and phylogenetic and conserved motifs. All members possess fully canonical motif structures of MAPK. Phylogenetic analysis indicated that *AcMAPK*s could be classified into five subfamilies, and these gene motifs in the same group showed high similarity. Gene structure analysis demonstrated that the number of exons in *AcMAPK* genes ranged from 2 to 29, suggesting large variation among kiwifruit *MAPK* genes. The expression profiles of these *AcMAPK* genes were further investigated using quantitative real-time polymerase chain reaction (qRT-PCR), which demonstrated that *AcMAPKs* were induced or repressed by various biotic and abiotic stresses and hormone treatments, suggesting their potential roles in the biotic and abiotic stress response and various hormone signal transduction pathways in kiwifruit. The results of this study provide valuable insight into the putative physiological and biochemical functions of *MAPK* genes in kiwifruit.

## 1. Introduction

Plants are often challenged by different biotic and abiotic stresses in nature, including pathogen infection, cold, drought, salt, and oxidative stresses; thus, they have developed some sophisticated signaling networks to sense and transmit environmental stimuli at the molecular or cellular levels [1]. A series of highly elaborate signaling networks are composed of some stress-activated molecular pathways [2]. Mitogen-activated protein kinase (MAPK) cascades play an important role in protein phosphorylation of signal transduction events and are one of the major mechanisms in controlling intracellular response to extra cellular signals in plants [3,4].

MAPK cascades are involved in the protein phosphorylation of signal transduction events that contribute to signaling [5], and MAPK cascades are classically composed of three protein kinases: MAPK (MAPK/MPK), MAPK kinase (MAPKK/MKK), and MAPK kinase kinase (MAPKKK/MAP3K/MEKK), but sometimes contain a MAPK kinase kinase kinase (MAPKKKK/MAP4K) that phosphorylates the corresponding downstream substrates [6,7,8]. MAPK can catalyze the phosphorylation of a substrate protein by chemically adding phosphate groups from adenosine triphosphate (ATP) [9]. MAP3Ks are the first component of this phosphorelay cascade, which phosphorylates two serine/threonine residues in a conserved S/T-X_3-5_-S/T motif of the MKK activation loop. Then, MKKs are dual-specificity kinases that activate the downstream MAPK through TDY or TEY phosphorylation motif in the activation loop (T-loop) [3,4,10]. The activated MAPK ultimately phosphorylates various downstream substrates, including transcription factors and other signaling components that regulate the expression of downstream genes [11]. MAPK proteins contain 11 evolutionary conserved kinase domains that may be involved in substrate specificity or protein–protein interaction [1,12]. 

Compared with MAPKKs and MAP3Ks, MAPKs act at the bottom of MAPK cascades in much greater numbers and show more complexity and sequence diversity. MAPK cascade proteins have TEY or TDY phosphorylation motifs in their activation loops between kinase domains VII and VIII, which provide protein-binding domains for the activation of MAPKs [3,6]. Plant MAPKs can be separated into four groups (A, B, C, and D) based on the phylogenetic relationships of the amino acid sequence and the phosphorylation motif. Members of the A, B, and C subfamily have the TEY motif at its phosphorylation site, and members of the D subfamily possess the TDY motif [3,4].

The MAPK proteins belong to a complex gene family in plants [13]. The identification and characterization of different members of the MAPK cascades have been revealed by genome sequencing projects in various plant species. The model plants that have been most studied are *Arabidopsis thaliana* and rice; there are 20 MAPKs in the *A. thaliana* genome [3], whereas the rice genome contains 17 MAPKs [14]. Recent research has reported that a total of 16, 19, 16, 14, 12, 17, 10, and 15 homologs in *MAPK* family genes have been identified from tomato (*Solanum lycopersicum*) [15], maize (*Zea mays*) [16], purple false brome (*Brachypodium distachyon*) [17], grapevine [13,18] and strawberry (*Fragaria vesca*) [19], tobacco (*Nicotiana tabacum*) [20], mulberry (*Moraceae morus*) [21], wheat (*Triticum aestivum*) [22] genomes, and as many as 21, 26, and 25 putative *MAPK* genes were identified in poplar (*Populus trichocarpa*) [23], apple (*Malus domestica*) [24], and banana (*Musa acuminata*), respectively.

In plants, MAPKs are involved in cellular responses to the regulation of the cell cycle, plant growth and development, hormones, and responses to biotic and abiotic stresses [7,25]. To date, several plant MAPK signaling cascades have been characterized in detail. The MEKK1-MKK4/5-MPK3/6 cascade was the first characterized signaling module in Arabidopsis, which up-regulated the expression of the transcription factors of WRKY22/29 and then increased resistance to both fungal and bacterial pathogens [25,26]. In addition, *AtMPK3* and *AtMPK6* are involved in the anther, embryo, inflorescence development, and stomatal distribution on the leaf surface [27,28]. The MEKK1-MKK1/2-MPK4 cascade was shown to positively regulate defense responses against necrotrophic fungi while negatively regulating defenses against biotrophic pathogens [29,30], also shown to be activated by drought, cold, and salt stresses [31]. *MAPK* genes in other important crops have also attracted considerable attention. For example, *OsMAPK3* and *OsMAPK6* are induced by a chitin elicitor in rice [32], *OsMPK5* is activated by pathogens and abiotic stresses [1], and overexpression of *OsMAPK33* enhances sensitivity to salt stress in rice through unfavorable ion homeostasis as negative regulators [33]. *ZmMPK3*, *ZmMPK5*, and *ZmMPK17* genes in maize are involved in signal transduction pathways associated with different environmental stresses [34,35,36]. Overexpression of *BnMAPK4* enhances resistance to *Sclerotinia sclerotiorum* in transgenic *Brassica napus* [37]. *GhMPK7* (*Gossypium hirsutum*) is induced by pathogen infection, and may be an important regulator in broad spectrum disease resistance and plant growth and development [38]. The expression of *VvMAPK3* and *VvMAPK6* genes were induced by salinity and drought [18].

Kiwifruit (*Actinidia chinensis*) is a nutritionally and commercially important and valuable fruit, well known for its remarkably high vitamin C content. For example, the Hongyang kiwifruit, which is derived from *A. chinensis* var. *chinensis* [39], is becoming a favorite of consumers, growers, and breeders due to its unique phenotype and high premium price at market. To date, systematic investigations and functional analyses of the MAPK gene family have not been reported for *A. chinensis*, despite the importance of MAPK proteins in multiple biological processes. Recently, the genome of a heterozygous kiwifruit cultivar “Hongyang” (*A. chinensis* var. *chinensis*) was sequenced [40], suggesting that kiwifruit has potential as a model organism for fruit trees. As such, it has become an imperative to compare the functions of gene families, particularly those having vital functions with the gene families characterized from *Arabidopsis* [41], which provides an opportunity for systematic analysis of *MAPK* in the kiwifruit species. With the rapid development of molecular biology and bioinformatics, the mining and positioning of functional genes in plant genome-wide data have become research hotspots. Due to the importance of MAPKs in diverse biological and physiological processes as well as their potential application to the development of improved stress tolerant transgenic plants, we performed the classification and phylogeny of the *MAPK* gene family of kiwifruit through bioinformatics analysis. Additionally, we conducted a comprehensive analysis of all the identified *AcMAPK* genes to determine which of these genes contribute to stress and hormone responses using quantitative real-time polymerase chain reaction (qRT-PCR) analysis. These data further provide information about the relationship between MAPK function and growth and development, disease resistance, and stress response of kiwifruit. The results of our identification and comprehensive investigation of the MAPK gene family in kiwifruit provide a theoretical basis for future gene cloning and expression, especially for the genetic improvement in the breeding of kiwifruit.

## 2. Results

### 2.1. Identification of MAPK Family Genes in Kiwifruit

To identify MAPK family genes from the *A. chinensis* genome, both Hidden Markov Model (HMM) and BLAST searches were performed using *Arabidopsis* and *Vitis. vinifera* MAPK proteins as query sequences. The comparison of the sequence of candidate proteins from BLAST and HMM hits were completed and 25 AcMAPK proteins were identified with top hits for *AtMAPK* and *VvMAPK* orthologs with an e-value cutoff of 1 × e^−50^. Then, some sequences were excluded because they encode very short polypeptides of amino acids, or did not contain the known conserved motifs of the MAPK family proteins by phylogenetic and conserved domains analysis. After multiple steps of screening and validation of the conserved domains, we finally identified 18 putative *AcMAPK* genes and the AcMAPK proteins were named according to the Gene ID number from the *A. chinensis* genome, designated as AcMAPK1–AcMAPK18 (Table 1), which was further supported by multiple sequence alignment analyses (Figure S1). The sequence data of all above *MAPK* genes are shown in Supplementary Material File 1. These putative *AcMAPK* genes were predicted to encode 336 (AcMAPK9) to 1056 (AcMAPK6) amino acids in length, with putative molecular weights (Mw) ranging from 38.82 kDa (AcMAPK12) to 119.46 kDa (AcMAPK6), and protein isoelectric points (pIs) ranging from 4.52 (AcMAPK11) to 9.82 (AcMAPK13). The subcellular localization was predicated and the putative AcMAPKs were located in the cytoplasm, nucleus, and chloroplast, except for AcMAPK9 and AcMAPK11, which were present in the peroxisome and vacuolar, respectively (Table 1).

The multiple sequence alignment data showed that all the putative AcMAPKs contain the classical TXY motif (Figure S1), which is located in the activation loop [6]. Moreover, MAPKs have a common docking (CD) domain that was observed in the extended C-terminal region, which is defined as (LH [D/E] XX [D/E] EPXC) and functions as a docking site for MAPKKs. Altogether, we identified 18 *MAPK* genes in kiwifruit.

To determine the chromosomal distribution of the identified AcMAPKs, the physical locations of the sequences of the 18 *AcMAPK* genes on the kiwifruit chromosomes were investigated. As shown in the location image (Figure S2 and Table 1), 18 genes were mapped on 11 chromosomes (including unknown chromosomes). Chromosomes 2, 11, 20, 23, 28, and 29 only contained one gene: *AcMAPK16*, *AcMAPK2*, *AcMAPK6*, *AcMAPK5*, *AcMAPK3*, and *AcMAPK14*, respectively. Chromosomes 13, 15, and 25 contained two genes, Chromosomes 1, and unknown contained three genes, respectively.

### 2.2. Phylogenetic Relationship Analysis of MAPK Gene in Kiwifruit

**Figure 1:**
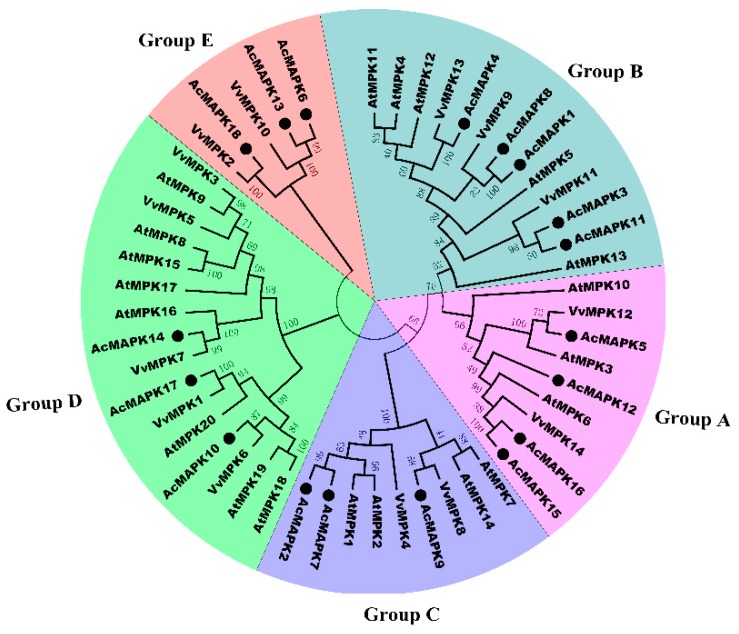
Phylogenetic relationship of putative *MAPK* genes in *Arabidopsis chinensis*, *V. vinifera*, and *A. thaliana*. The phylogenetic tree was created using MEGA5.0 program with the neighbor-joining (NJ) method. Bootstrap values for 1000 replicates are indicated at each branch. Letters A–E indicate different groups of MAPKs.

In order to evaluate the evolutionary relationships among the MAPK proteins, a phylogenetic tree was constructed with amino acid sequences of 18 putative *AcMAPKs* from kiwifruit, 20 *AtMAPKs* from Arabidopsis, and 14 *VvMAPKs* from grapevine. In plants, MAPK proteins have diverged into four major subfamilies (A, B, C, and D) [3], as shown in Figure 1. The phylogenetic analysis showed that the 18 putative *AcMAPKs* could be divided into five distinct groups (groups A, B, C, D, and E) together with their MAPK orthologs in *Arabidopsis* and grapevine, which are more groups than identified in previous reports [42]. *AcMAPKs* belonging to the A, B, C, and E subfamilies all possess a TEY motif, except for *AcMAPK18*, which harbors a TDY motif, whereas the D subfamily possesses a TDY motif at the activation site (Table 1). 

*AcMAPK5*, *AcMAPK12*, *AcMAPK15* and *AcMAPK16* genes are clustered in Group A, which contains well-characterized *MAPK* genes including *AtMPK3*, *AtMPK6*, *VvMPK12*, and *VvMPK14* genes. *AcMAPK1*, *AcMAPK3*, *AcMAPK4*, *AcMAPK8*, and *AcMAPK11* genes belong to Group B, which includes *AtMPK4*, *AtMPK5*, *AtMPK11*, *AtMPK12*, *VvMPK9*, and *VvMPK11* genes. Group C contained three genes: *AcMAPK2*, *AcMAPK7*, and *AcMAPK9* genes. Group D includes *AcMAPK10*, *AcMAPK14*, and *AcMAPK17* genes of the kiwifruit MAPKs (Figure 1), which have a TDY motif, consistently found in members of the other MAPK subfamily. *AcMAPK6*, *AcMAPK13*, and *AcMAPK18*, genes belonging to group E, were separated from other groups (Figure 1). 

### 2.3. Gene Structure Analysis of MAPK Gene in Kiwifruit

**Figure 2:**
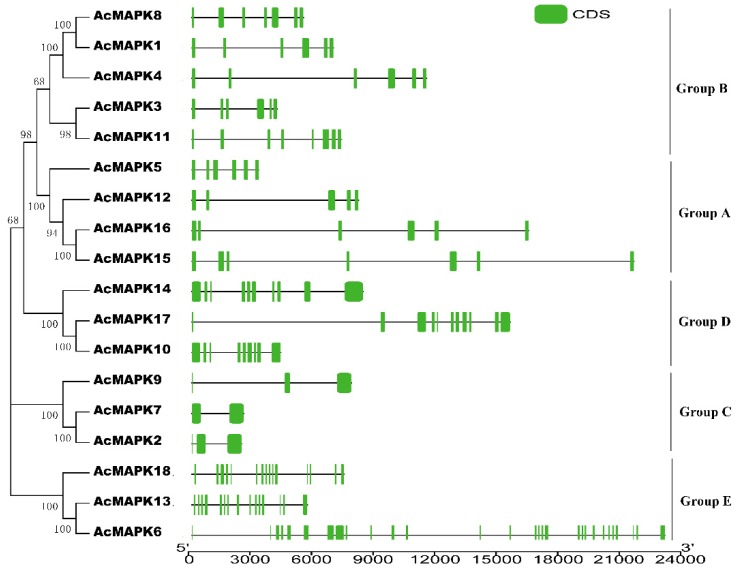
The phylogenetic analysis and intron/exon structures of putative *MAPK* genes in *A. chinensis*. The phylogenetic tree (left panel) was created using MEGA5.0 program with the neighbor-joining (NJ) method. Exon/intron structures of the *MAPK* genes are shown in the right panel. The green boxes indicate the exons, whereas the single lines indicate introns. Gene models were drawn to scale as indicated on bottom.

The identification of exon-intron structures for each *AcMAPK* gene was determined by aligning corresponding genomic DNA sequences. The exon/intron structures of putative *AcMAPK* genes could also be divided into five subgroups based on their phylogenetic relationship (Figure 2). We found that *AcMAPK* genes in different groups have strikingly different exon/intron structures, but that the gene structures of putative *AcMAPK* members in the same group were highly conserved in kiwifruit (Figure 2). The putative *AcMAPK* members were composed of exons varying from five to seven in Group A. Group B contains exons varying from six to eight, whereas those of Group C only had two or three exons. Nine to 11 exons were present in the *AcMAPK* genes in Group D, and Group E had a larger number of exons with variable exon lengths than other groups; in this group, the number of exons varied from 16 to 29 (Figure 2). 

### 2.4. The Conserved Motifs Domain and Promoter Regions Analysis of MAPK Gene in Kiwifruit

**Figure 3:**
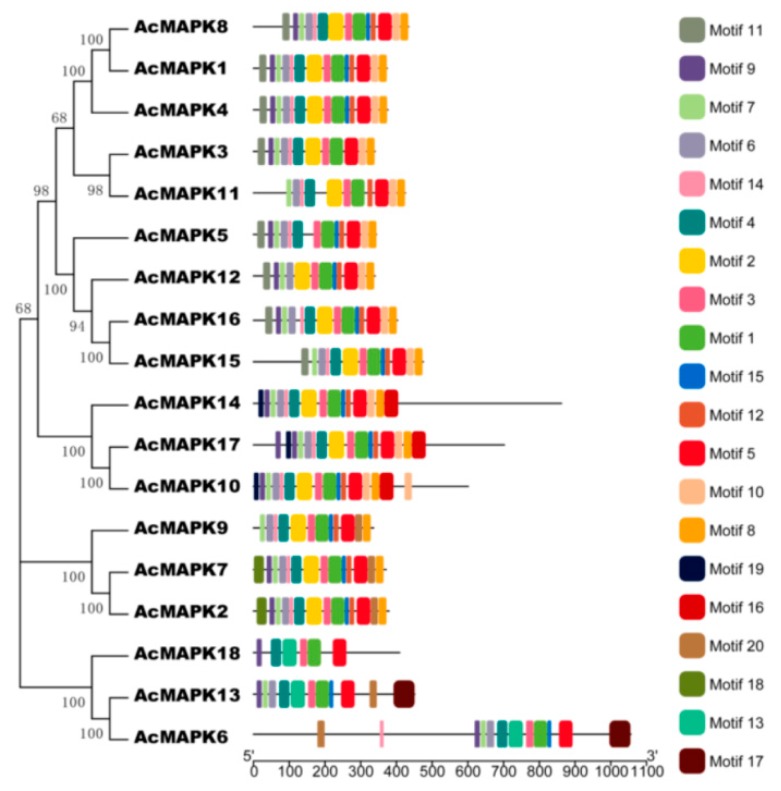
The conserved motifs of kiwifruit putative MAPKs according to the phylogenetic relationship. All motifs were identified online with the MEME program with the complete amino acid sequences of the 18 MAPKs. Different colors of the boxes represent different motifs in the corresponding position of each AcMAPK proteins. Detailed information of the 20 motifs is provided in Supplementary Material File 2.

To explore the structural diversity of the *AcMAPK* genes, we submitted the 18 putative AcMAPK protein sequences to the online MEME program to search for conserved motifs (Figure 3, Supplementary Material File 2) [43]. As shown in Figure 3, 20 conserved motifs were identified. Specifically, all the identified AcMAPKs contained motifs 1, 3 (contained the TXY signature motif), and 5 (Figure 3), indicating that all the kiwifruit MAPKs were typical of the MAPK family. Additionally, the majority of AcMAPKs contained the 13 protein kinase motifs (motifs 1, 2, 3, 4, 5, 6, 7, 8, 9, 10, 12, 14, and 15) (Figure 3). We found all the members identified in the same subfamily shared similar conserved motifs. For instance, along with all the conserved motifs, most MAPK proteins in Groups A and B had specific motif 11 at the N-terminal region, whereas 18 motifs only existed in most MAPKs in Group C. MAPKs in group D contained specific motif 19 at the N-terminal region as well as motif 16 at the C-terminal region, and motifs 13 and 17 only existed in Group E of the MAPK proteins (Figure 3). 

To further investigate the potential functions and transcriptional regulation of these putative *AcMAPK* genes, we identified the *cis*-regulatory elements by the transcriptional start site (ATG) using 1500 bp upstream regions. We found a large amount of pathogen-related, stress-related, and hormone-related *cis*-elements in the putative promoter regions of the putative *AcMAPK* genes in kiwifruit. Some genes contain more *cis*-elements, and some genes contain less (Figure S3, Supplementary Material File 3).

### 2.5. Expression Profiles of AcMAPK Genes in Response to Hormone Treatments

**Figure 4:**
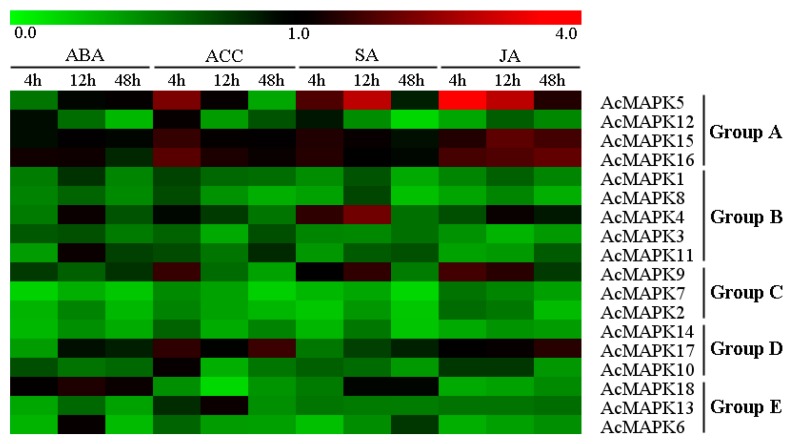
Hierarchical clustering of the expression profiles of *AcMAPK* genes in response to different hormones treatments in kiwifruit leaves. ABA: treatments with abscisic acid, ACC: treatments with 1-Aminocyclopropanecarboxylic Acid, SA: treatments with salicylic acid; JA: treatments with jasmonic acid, details of the treatments are reported in Materials and Methods. The heat-map demonstrates the relative fold-change expression for all *AcMAPK* genes in response to the different hormone treatments in comparison to their respective controls. Red and green colors represent increased or decreased expression levels, respectively, in comparison to controls, as reported by the scale. Genes were clustered according to phylogenetic relationships in expression profiles. Relative expression values for each gene and each treatment are provided in Figures S4 and S5.

To investigate the contribution of *AcMAPK* to various hormone treatments, we subjected four-week-old seedlings of Jinkui (*A. chinensis* var. *deliciosa*) to examine the expression patterns of 18 *AcMAPK* genes using quantitative real-time PCR. In order to obtain a comprehensive view and compare the effects of different treatments on a given gene, the produced heat-map graphic of the expression profiles for all genes and all hormone treatments is provided in Figure 4. It was interesting that the transcript levels of almost all genes were down-regulated in response to hormone treatments (Figure 4, Figure S4, and Figure S5). In our work, the transcript levels of all *AcMAPK* genes were down-regulated after abscisic acid (ABA) treatment (Figure 4 and Figure S4A). *AcMAPK5*, *AcMAPK 9*, *AcMAPK15*, and *AcMAPK16* genes were up-regulated at four hours. *AcMAPK17* showed obvious up-regulation at 4 and 48 h after 1-aminocyclopropanecarboxylic acid (ACC) treatment (Figure 4 and Figure S4B). These genes (*AcMAPK4*, *AcMAPK5*, and *AcMAPK9*) were significantly up-regulated at 12 h after salicylic acid (SA) treatment, and *AcMAPK5*, *AcMAPK9*, *AcMAPK15*, *AcMAPK16*, and *AcMAPK17* were induced by jasmonic acid (JA) treatment (Figure 4 and Figure S5). In these genes, *AcMAPK5* demonstrated significantly higher induction after the hormone treatments than other genes.

### 2.6. Expression Patterns of AcMAPK Genes under Abiotic and Biotic Stresses

**Figure 5:**
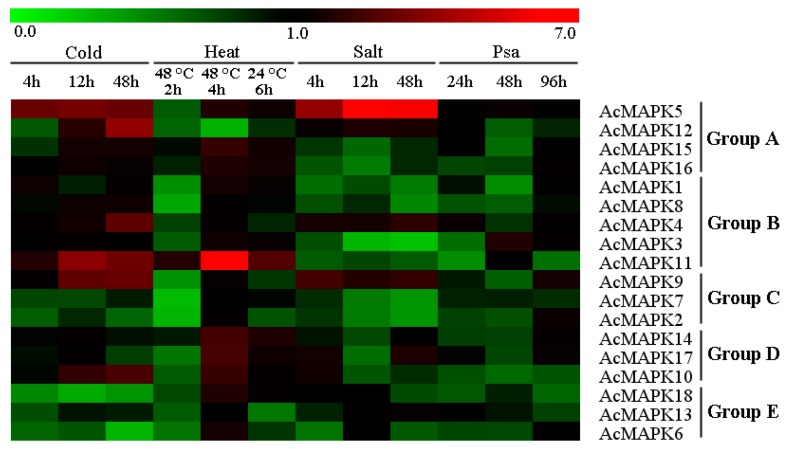
Hierarchical clustering of the expression profiles of *AcMAPK* genes in response to various biotic and abiotic stresses. Cold: treatment at 4 °C; Heat: treatment at 48 °C and 24 °C; Salt: treatment with NaCl; Psa: *Pseudomonas syringae* pv. *actinidiae* infection. Details of the treatments are reported in Materials and Methods. The heat-map depicts the fold-change of the relative expression of all *AcMAPK* genes in response to the various treatments in comparison to their respective controls. Red and green colors represent increased or decreased expression levels, respectively, in comparison to controls, as reported by the scale. Genes were clustered according to phylogenetic relationships in expression profiles. Relative expression values for each gene and each treatment are provided in Figures S6 and S7.

We also investigated the expression of *AcMAPK* genes in response to various abiotic and biotic stress responses with different hormone treatments (Figure 5, Figure S6, and Figure S7). In response to cold stress, the expression of five *AcMAPK* genes (*AcMAPK5*, *AcMAPK9*, *AcMAPK10*, *AcMAPK11*, and *AcMAPK12*) were significantly up-regulated throughout the treatment process, and *AcMAPK4* was up-regulated at 48 h of treatment; whereas *AcMAPK2*, *AcMAPK6*, *AcMAPK7*, *AcMAPK13*, and *AcMAPK18* genes were down-regulated at all treated time points (Figure 5 and Figure S6A). After heat treatment, nine *AcMAPK* genes (*AcMAPK1*, *AcMAPK5*, *AcMAPK10*, *AcMAPK11*, *AcMAPK14*, *AcMAPK15*, *AcMAPK16*, *AcMAPK17* and *AcMAPK18*) were up-regulated after four hours of heat stress treatment at 48 °C. The *AcMAPK11* gene was significantly up-regulated (Figure 5 and Figure S6B). With salt treatment, the expression of *AcMAPK4*, *AcMAPK5*, *AcMAPK9*, and *AcMAPK12* genes were significantly up-regulated at all treatment time points, and *AcMAPK10*, *AcMAPK13* and *AcMAPK17* genes were up-regulated at several treated time points, whereas the remaining genes were almost down-regulated under salt treatment (Figure 5 and Figure S7A). Almost all the *AcMAPKs* genes (except *AcMAPK2*, *AcMAPK3* and *AcMAPK9*) were down-regulated after *Pseudomonas syringae* pv. *actinidiae* (Psa) treatment (Figure 5 and Figure S7B).

## 3. Discussion

In recent years, the characterization of gene families has been useful for studying their function [44]. The accuracy and reliability of gene family evolutionary characterization analysis depend on the genomic sequences. The availability of the complete kiwifruit genome sequence has made it possible to identify all the MAPK family members in this plant species for the first time. In this study, we identified 18 putative *MAPK* genes in the *A. chinensis* genome. The numbers are comparable to those in *A. thaliana* genome, where 20 MAPK members have been identified [3], but the genome size of *A. chinensis* (~616.1 Mb) is approximately four times that of the *A. thaliana* genome (~125 Mb). The 18 members in kiwifruit is a larger number than found in grapevine (14 members) and strawberry (12 members), but smaller than in apple (26 members) and banana (25 members) in fruit. The full-length sequences of putative *AcMAPK* ranged from 336 to 1056 amino acids. Variation in the length of the entire *MAPK* gene is usually due to differences in the length of the MAPK domain or the number of introns [18]. We found most members in the same group share a similar exon/intron structure, which was similar to other plants, including Arabidopsis, tomato, and poplar [1,7,15]. So, the exon/intron structures of each gene cluster originated from tandem or segmental duplication events in the *MAPK* gene family and tended to share similar structure organizations, except for tiny differences. The results were consistent with those of domain and phylogenetic analyses performed.

In plants, *MAPK* genes have diverged into four subfamilies based on the conserved residues of the TEY/TDY motifs in the activation loop region (T-loop) [3]. However, phylogenetic analysis showed that the 18 putative *AcMAPKs* were divided into five distinct groups (A, B, C, D, and E), together with their MAPK orthologs in *Arabidopsis* and grapevine, which is more than previously reports [42]. *AcMAPK5*, *AcMAPK12*, *AcMAPK15*, and *AcMAPK16* belong to Group A, which contains *AtMPK3* and *AtMPK6* (Figure 1). It has been well-characterized that *AtMPK3* is activated in response to pathogens and abiotic stresses, and *AtMPK6* can be activated by various abiotic and biotic stresses [1]. *AcMAPK1*, *AcMAPK3*, *AcMAPK4*, *AcMAPK8*, and *AcMAPK11* belong to Group B, which includes *AtMPK4*, *AtMPK5*, *AtMPK11*, *AtMPK12*, *VvMPK9*, and *VvMPK11* (Figure 1). The MAPKs in Group B are involved in both abiotic stress responses and cell division in Arabidopsis. *AtMPK4* and its upstream *AtMKK2* can be activated by biotic and abiotic stresses [31]. Group C contained three genes: *AcMAPK2*, *AcMAPK7*, and *AcMAPK9* (Figure 1). Members from this group in other plant species are known to be regulated by both biotic and abiotic stresses. For example, *AtMPK1* in Group C is regulated by salt stress treatment [4], and *AtMPK1* and *AtMPK2* are activated by ABA [45]. In addition, the rice *BWMK1* and alfalfa *TDY1* genes in Group C are activated by wounding and pathogens [46]. Group D includes *AcMAPK10*, *AcMAPK14*, and *AcMAPK17* of the kiwifruit MAPKs (Figure 1), which have the TDY motif in their T-loop, which are consistently found in members of the other MAPK groups. We found that Group D is the largest group of MAPKs in most plant species. *AcMAPK6*, *AcMAPK13*, and *AcMAPK18*, belonging to Group E, were separated from other groups (Figure 1). The *AcMAPKs* genes of Group E are found only in the grapevine genome among other plant species; there were no orthologs of *AtMAPK* in *A. thaliana*.

The result of our examination of the conserved motifs domain found all the identified *AcMAPKs* contained motifs 1, 3 (contained the TXY signature motif), and 5 (Figure 3), indicating that all the kiwifruit MAPKs were typical of the MAPK family. Above, we stated that all members identified in the same subfamily shared similar conserved motifs. For instance, along with all the conserved motifs, most MAPK proteins in Groups A and B had specific motif 11 at the N-terminal region, whereas 18 motifs only existed in most MAPKs in Group C. The MAPKs of Group D contained the specific motif 19 at the N-terminal region as well as motif 16 at the C-terminal region, and motifs 13 and 17 only existed in Group E of the MAPK proteins (Figure 3). This suggests functional consistency among the MAPK members in the same group. Moreover, motifs in each group were diverse, in accordance with the intron/exon structure of each group. Thus, the composition and the sequential order of these motifs in the same group showed high similarity. A large amount of stress-, pathogen-, and hormone-related *cis*-elements were found in the putative promoter regions of the *AcMAPK* genes in kiwifruit as shown by *cis*-regulatory elements analysis. The existence of these *cis*-elements suggested that these *AcMAPK* genes might have potential functions in various stress signaling pathways. Similar *cis*-elements were found in *MAPK* genes of tomato [15] and *B. distachyon* [17].

A large number of reports demonstrated the involvement of *MAPK* genes in response to various biotic and abiotic stresses and hormone signaling [4]. AtMAPK3 and AtMAPK6 of Group A are the most prominent kinases, which have been widely studied and have been strongly associated with various environmental stresses in *Arabidopsis* [11]. In this study, he transcription level of the *AcMAPK5* gene, which is the kiwifruit orthologue of the *AtMAPK3* gene, showed an obvious up-regulation response to cold, heat, salt, ACC, SA, and JA treatments, which indicates that *AcMAPK5* might be an important regulator in response to abiotic stresses and hormone signaling molecules. Notably, the gene expression down-regulation of *AcMAPK12* observed in any hormone treatment was induced transcriptionally by cold and salt stress, suggesting that activation of AcMAPK12 protein kinase activities might be not correlated with their transcript levels, similar to AtMAPK6. However, the expression of *AcMAP15* and *AcMAP16* genes was repressed by most hormone (except for JA) and heat treatments; these results are similar to previous reports in which the MKK3/MPK6 module was proposed to participate in JA signaling [47]. The MAPKs of Group B (*AtMAPK4* and *AtMAPK11*) have been implicated in pathogen defense and abiotic stress responses [48]. The relationship of MAPK signaling pathways and SA in plant abiotic stress responses was recently characterized [49]. The *AcMAPK11* gene showed an obvious up-regulation response to cold and heat stress. The *AcMAPK4* gene was induced by cold, salt, SA, and ABA treatments, suggesting the involvement of these genes in abiotic stress tolerance and hormone signal transduction in kiwifruit. The expression of *AcMAPK* genes from Group B was repressed by most stresses and hormone treatments, suggesting that *AcMAPK* genes of Group B may function in an early stage of stress signaling transduction as negative regulators in kiwifruit. The MAPKs of Group C in *Arabidopsis* are activated by ABA, providing evidence for a role in an ABA-induced MAPK pathway in plant stress signaling [50]. However, the transcription level of *AcMAPK2*, *AcMAPK7*, and *AcMAPK9* from Group C were down-regulated by ABA treatment in this study, which suggests that the involvement of these genes in ABA signaling might be regulated at the level of translation. The *AcMAPK9* gene was induced by cold, heat, salt, *P. syringae*, ACC, SA, and JA treatments, which suggests that this gene might also have important functions in abiotic stress and hormone signaling. *AtMPK7* was significantly up-regulated in response to cold stress [17]. *AcMAPK9*, which showed the highest homology to *AtMPK7*, showed strong activation by cold stress, suggesting a similar function. The *MAPK* genes of Group D have not been as well studied as those of Groups A and B. *AcMAPK10* and *AcMAPK17* genes in Group D were induced by cold, heat, salt, and ACC treatments. It is interesting that expression of *AcMAPK14* was up-regulated by all biotic and abiotic stresses, whereas down-regulation induced by all hormone treatments. Together, these results indicate possible roles of the *MAPK* genes of Group D in abiotic stress responses and hormone signaling. The *AcMAPK*s genes of Group E are found only in the grapevine genome among other plant species; there were no orthologs of *AtMAPK* in *Arabidopsis*. The expression of Group E gene members was repressed by most biotic and abiotic stresses, similar to their response to hormone treatments, except for heat treatment. However, more research is needed to determine the specific functions of the MAPK family of genes by additional experiments.

## 4. Materials and Methods

### 4.1. Genome-Wide Identification of MAPK Genes in Kiwifruit

For identification of the *MAPK* gene family, the sequences of *Arabidopsis* MAPK cascade proteins were obtained from TAİR (https://www.arabidopsis.org/). The MAPK protein sequences of grapevine were obtained from the *V. vinifera* proteome 12× database (http://www.genoscope.cns.fr/externe/GenomeBrowser/Vitis/). Kiwifruit (*A. chinensis*) assembly and annotation were downloaded from kiwifruit genome database (http://bioinfo.bti.cornell.edu/cgi-bin/kiwi/download.cgi). These sequences were used as queries to search against the kiwifruit protein databases by the BLASTP program with an e-value of 1 × e^−50^ as the threshold. The local Hidden Markov Model-based searches (HMMER: http://hmmer.janelia.org/), built from all the known MAPK protein sequences from *Arabidopsis* and grapevine, were used to identify the *MAPK* genes in kiwifruit. To identify predicted *AcMAPK* genes accurately from genome sequences, the unique sequences obtained from the above-mentioned programs were further filtered based on the typical structural features of plant MAPK proteins as previously reported [1,13]. The *AcMAPK* genes were accepted only if they contained the essential TDY or TEY signature motif and the 11 conserved subdomains.

### 4.2. Sequence Alignment, Phylogenetic Analysis, Chromosomal Location, and Gene Structure Construction

The protein theoretical molecular weight and isoelectric point were predicted using compute pI/MW (http://au.expasy.org/tools). Multiple alignments of the nucleotide and amino acid sequences were performed using ClustalW [51]. The phylogenetic analysis was constructed based on the sequences of MAPK proteins from *Arabidopsis*, *V. vinifera*, and kiwifruit using a neighbor-joining (NJ) method with 1000 bootstrap replicates and visualized with MEGA5 software [52]. The chromosomal distribution of all *AcMAPK* genes was determined based on the results of identification, and subsequently the location images of *AcMAPK* genes were drawn with MapInspect software (http://www.softsea.com/review/MapInspect.html). The exon/intron structure analysis of the *AcMAPK* genes was conducted and displayed by comparing CDSs and their corresponding gene sequences from genomic using the Gene Structure Display Serve [53]. The MEME program was used to statistically identify conserved motifs in the complete amino acid sequences of AcMAPK proteins [43].

### 4.3. Cis-Element Analysis of Putative Promoter Regions

To investigate *cis*-elements in the promoter regions of the identified genes, we downloaded the genomic DNA sequences upstream from the kiwifruit database to search the initiation codon (ATG) of each gene [54]. The putative *cis*-regulatory elements in the promoter regions sequences were analyzed via the PLACE database (http://www.dna.affrc.go.jp/ PLACE/).

### 4.4. Plant Materials and Treatments

The kiwifruit cultivar “Jinkui” (*Actinidia chinensis* var. *deliciosa*) were maintained in vitro on Murashige and Skoog (MS) medium supplied with 6-benzylaminopurine (6-BA, 3.0 mg/L, Sigma-Aldrich, St. Louis, MO, USA), and naphthalene acetic acid (NAA, 0.2 mg·L^−1^, Sigma) under a 16/8 h photoperiod (100 µmol m^−2^·s^−1^) at 25 °C in a growth chamber. Four-week-old plants were used for hormones, freezing (4 °C), and heat stress treatments. Shoots with good growth vigor were collected from Jinkui kiwifruit trees and cultured in MS medium, and maintained in growth chambers, then used for *Pseudomonas syringae* pv. *actinidiae* (Psa) treatments. The conditions included a temperature of 25 °C and 12/12 h light/dark cycles. Two-year-old Jinkui cutting seedlings, which were used for salt treatment, were grown in nutrient soil in a greenhouse at a temperature of 25–28 °C during the day and 20–25 °C during the night.

Several of the stress treatments were performed in kiwifruit as described previously [55]. For treatments with abscisic acid (ABA), 1-aminocyclopropanecarboxylic acid (ACC), salicylic acid (SA) and jasmonic acid (JA), plants with eight fully expanded leaves per tissue-culture container (240 mL) were sprayed with 0.01 mM ABA, 0.01 mM ACC, 0.1 mM SA, and 0.02 mM JA. All the chemicals were purchased from Sigma-Aldrich and dissolved in sterile distilled water. The leaves were harvested at 0, 4, 12, and 48 h post-treatment. For cold stress, seedlings were grown at 4 °C for 0, 4, 12, and 48 h. For heat stress, seedlings were grown at 48 °C for 0, 2, and 4 h, and then at 24 °C for another 6 h. For salt stress, the cutting seedlings were soaked at high salinity (200 mM NaCl) for 0, 4, 12, and 48 h. The seedling leaves and seedling cuttings from both treated and control plants were harvested in the above treatments. For *Pseudomonas syringae* pv. *actinidiae* (Psa) bacterial infection, bacterial cells were suspended in distilled water and adjusted to an OD_600_ = 0.2, and injected into the seedling stems, which were carved with a knife. Only carved seedling stems were used as the control (CK), inoculated with Psa, and sampled at 24, 48, and 96 h. Every treated sample had a corresponding regularly-watered control. Three biological replicates were collected per time point, each comprising five independent plants. All samples were immediately frozen in liquid nitrogen and stored at −80 °C. 

### 4.5. Total RNA Isolation and qRT-PCR Expression Analysis

Total RNA was extracted from the collected samples as described previously with some modifications [5]. Reverse transcription of mRNA was synthesized with a Prime Script™ RT Reagent Kit (Perfect Real Time, TaKaRa, Ostu, Japan) with 1 µg total RNA. The cDNA samples were diluted 1:10 with sterile double-distilled water and stored at −20 °C before being used.

The expressions of *AcMAPKs* were examined by qRT-PCR using a SYBR Green method on an ABI 7300 Real-time PCR System (Applied Biosystems, Waltham, MA, USA). The primer sequences used were designed based on gene sequences and the Beacon designer software (NJ, USA), as shown in Supplementary Material File 4 in this study. Kiwifruit actin was used as the housekeeping gene to monitor cDNA abundance [56]. qRT-PCR was carried out as described previously [55]. The relative gene expression level was calculated according to the 2^−ΔΔ*C*t^ method, where ∆∆*C*_t_ = (*C*_t target gene_ − *C*_t actin_)_treatment_ − (*C*_t target gene_ − *C*_t actin_)_ck_ [5,57]. To visualize the relative expression levels data, 0 h at each treatment was normalized as “1”, which are presented as the mean fold changes between treated and control samples at each time point ± standard deviations (SDs). The expression data of the 18 *AcMAPK* genes were transformed in log_2_ values and used for heat map generation. The heat map was created with MeV4.8 software (Boston, MA, USA) (http://www.tm4.org/mev/).

### 4.6. Statistical Analysis

Statistical analyses were performed using SPSS version 17.0 software (Chicago, MI, USA) and Excel. All results of expression data are indicated as means ± standard deviations (SDs), and the level of significance between different time points was set at *p* < 0.05. 

## 5. Conclusions

Kiwifruit (*Actinidia chinensis*) has become an important commercial fruit due to its pleasant flavor and nutritional components that benefit human health [39]. However, research progress on kiwifruit has been relatively slow compared to grape and apple horticultural crops. MAPK cascade is one of the major pathways in plants, with MAPK as the downstream molecule of the MAPK cascade playing an important role in singling [6]. In this study, we identified 18 putative *MAPK* genes from the kiwifruit genome and established their classification and phylogenetic relationships, gene structure, conserved protein domains/motifs, and promoter regions. The phylogenetic relationship of MAPKs among kiwifruit demonstrated that the 18 *AcMAPK* genes were grouped into five subgroups (A, B, C, D, and E), and most genes within the same group generally share similar exon/intron patterns and conserved protein domains and motifs. Our analyses strongly supported the identity of each subgroup. The expression profiles of the MAPK cascade genes in various biotic and abiotic stresses and hormones treatments were discussed. The majority of the MAPK cascade genes could be induced by one or more specific treatments. In summary, our study provides an overview of the *MAPK* gene family in kiwifruit, which will be helpful in the biochemical functional characterization of the MAPK cascades in kiwifruit.

## Figures and Tables

**Table 1:** The characteristics of putative *MAPK* genes in kiwifruit.

Name	Gene ID	Chromosome	Length of Protein in AA (Amino Acid)	CDS (Coding Sequences) Length in bp	MW (Molecular Weights) (kDa)	PI (Protein Isoelectric Points)	Number of Exons	T-Loop	Subcellular Location
*AcMAPK1*	Achn005721	15	374	1125	42.93	6.44	6	TEY	Chloroplast
*AcMAPK2*	Achn025711	11	379	1140	43.65	7.12	3	TEY	Nuclear, Cytoplasm
*AcMAPK3*	Achn060571	28	340	1023	38.99	4.89	6	TEY	Cytoplasm
*AcMAPK4*	Achn074341	Un	376	1131	43.36	6.51	6	TEY	Cytoplasm
*AcMAPK5*	Achn082251	23	344	1035	39.74	5.41	6	TEY	Cytoplasm
*AcMAPK6*	Achn098501	20	1056	3171	119.46	9.14	29	TEY	Cytoplasm
*AcMAPK7*	Achn131961	Un	371	1116	42.56	6.89	2	TEY	Cytoplasm
*AcMAPK8*	Achn132381	25	434	1305	49.42	7.39	7	TEY	Cytoplasm
*AcMAPK9*	Achn135551	1	336	1011	39.11	7.90	3	TEY	Peroxisome
*AcMAPK10*	Achn146591	13	601	1806	67.76	9.17	9	TDY	Chloroplast
*AcMAPK11*	Achn195331	13	425	1278	48.30	4.52	8	TEY	Vacuolar
*AcMAPK12*	Achn209161	1	340	1023	38.82	6.25	5	TEY	Cytoplasm
*AcMAPK13*	Achn228801	25	451	1356	51.08	9.82	16	TEY	Cytoplasm, Nuclear
*AcMAPK14*	Achn237151	29	862	2589	97.02	9.25	10	TDY	Nuclear, Chloroplast
*AcMAPK15*	Achn248791	Un	475	1428	54.89	5.15	7	TEY	Cytoplasm
*AcMAPK16*	Achn252431	2	403	1212	46.22	5.69	6	TEY	Cytoplasm
*AcMAPK17*	Achn296271	1	702	2109	79.02	9.03	11	TDY	Chloroplast
*AcMAPK18*	Achn377281	15	409	1230	46.34	5.40	16	TDY	Nuclear

## Supplementary Materials

Supplementary materials can be found at http://www.mdpi.com/1422-0067/19/9/2510/s1.
