# Supplementary material for: Genome-Wide Bioinformatics Analysis of MAPK Gene Family in Kiwifruit (Actinidia Chinensis)
Source: Int J Mol Sci. 2018 Aug 24;19(9):2510. doi: 10.3390/ijms19092510 (PMC6164783; doi:10.3390/ijms19092510)
Supplement: Supplementary file 1 [file ijms-19-02510-s001.zip › ijms-336521 supplementary figures.docx]

**Genome-Wide Bioinformatics Analysis of *MAPK* gene Family in Kiwifruit (*Actinidia Chinensis*)**

Gang Wang, Tao Wang, Zhan-Hui Jia, Ji-Ping Xuan, De-Lin Pan, Zhong-Ren Guo and Ji-Yu Zhang *


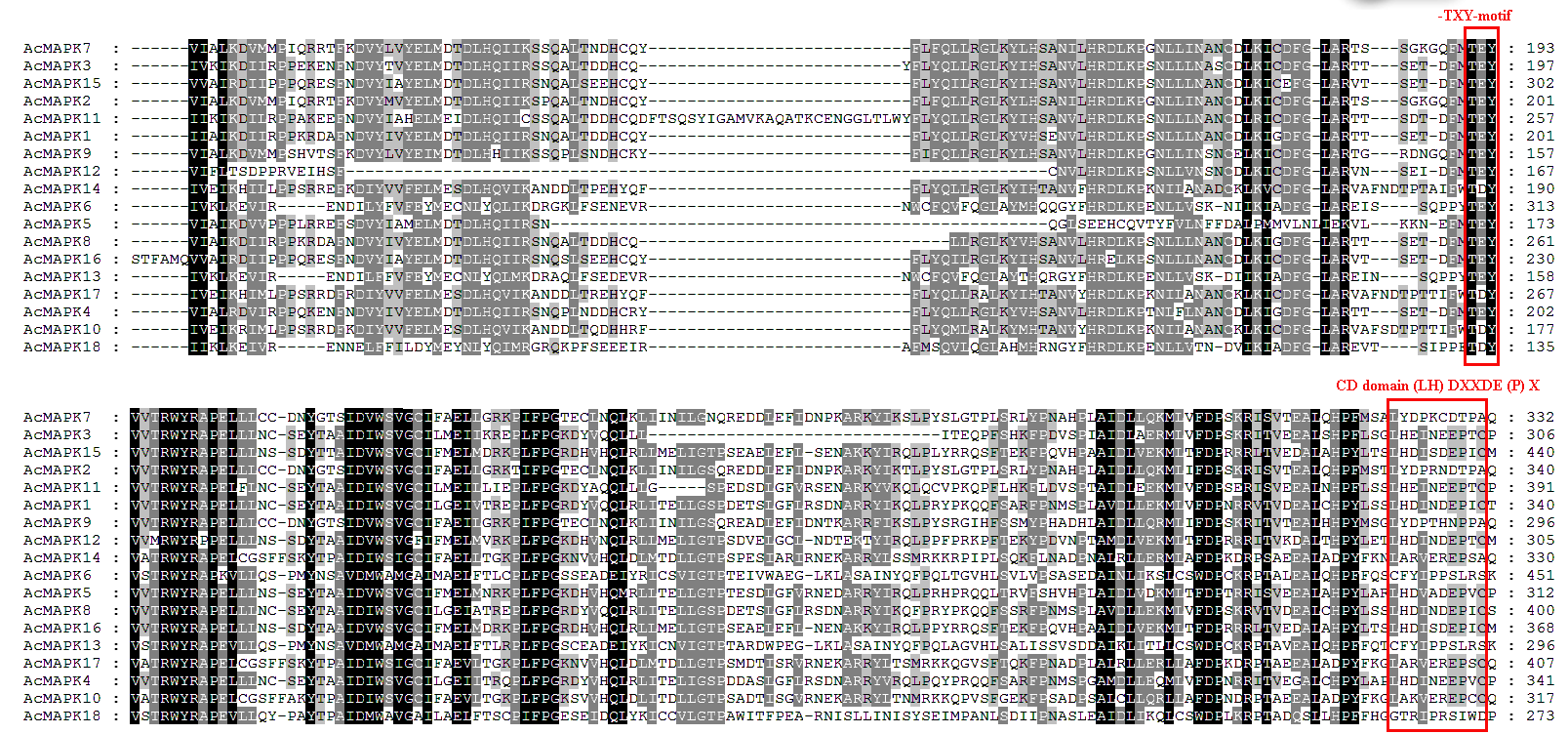


**Figure S1.** Sequences alignment analysis of the putative AcMAPK proteins in *A. chinensis*. Alignment was performed using ClustalW and displayed using GeneDoc. The conserved amino acid signature motif TXY and common docking domain are highlighted in red box above the alignments.


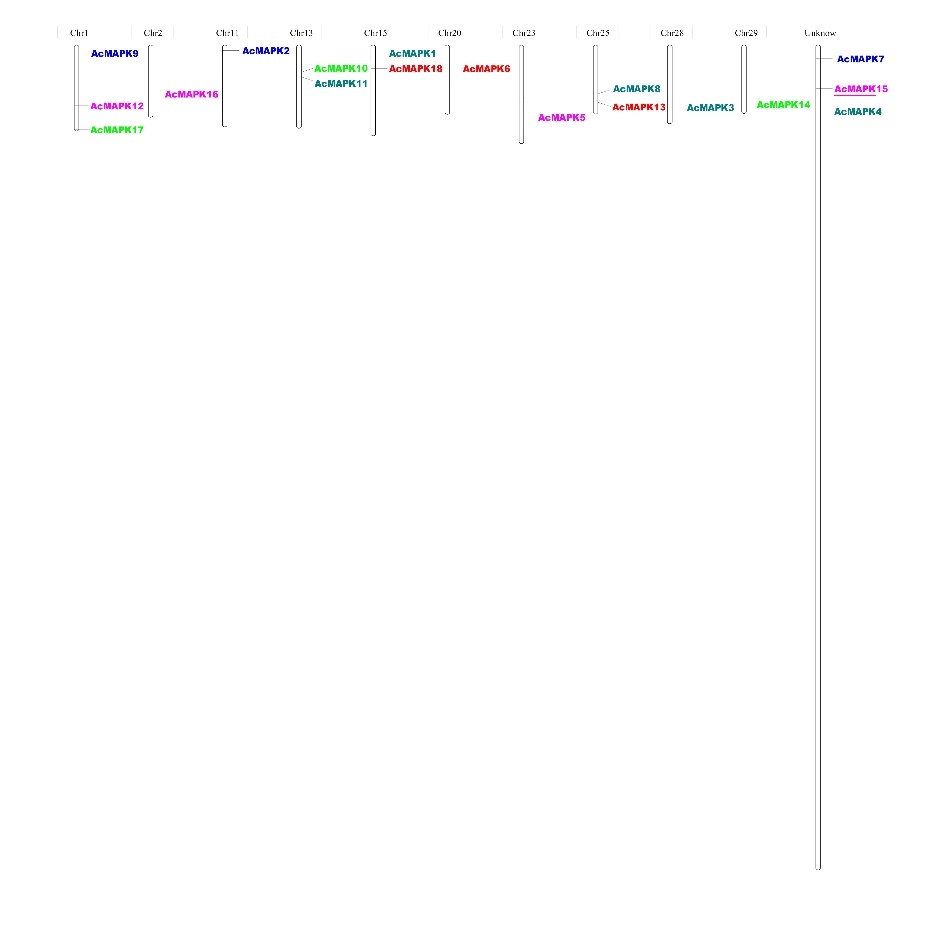


**Figure S2.** Chromosomal locations of 18 putative AcMAPK proteins in *A. chinensis*. The gene names are on the right side of each chromosome according to the approximate physical location of each *AcMAPK* gene, *AcMAPKs* genes are in same subgroups are shaded corresponding colors.


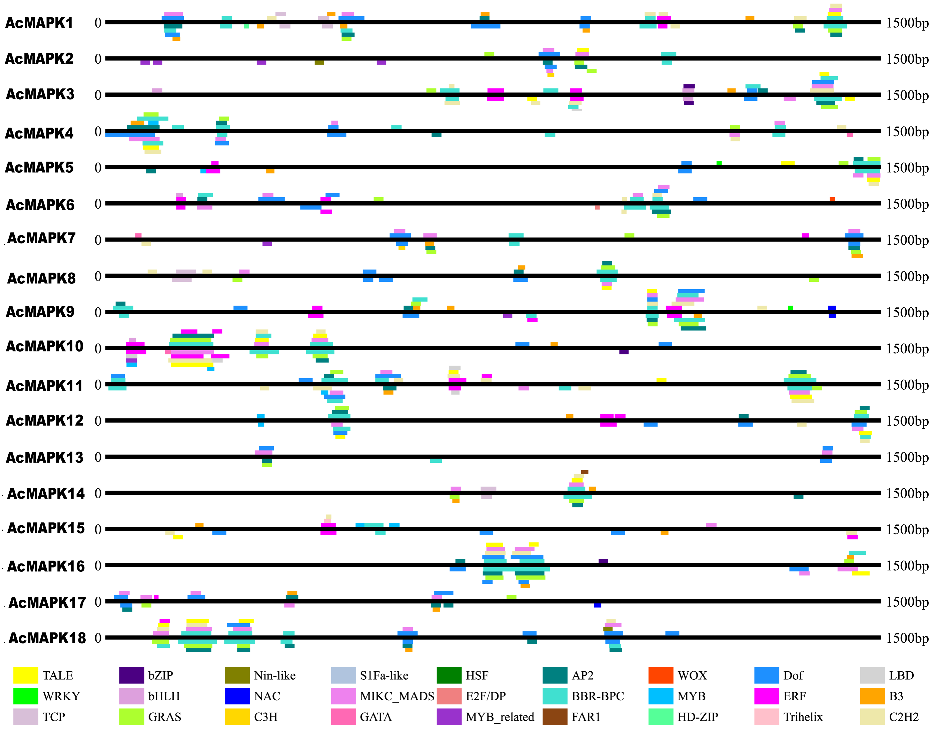


**Figure S3.** The *cis*-elements in promoter sequences of *AcMAPK* genes in *A. chinensis*. The elements in the promoter sequences were analyzed via the PLACE database, and different colors of the boxes represent different promoter elements in the corresponding position of each AcMAPK proteins. The detailed information of 27 promoter elements was illustrated in Supplementary Material file 3.


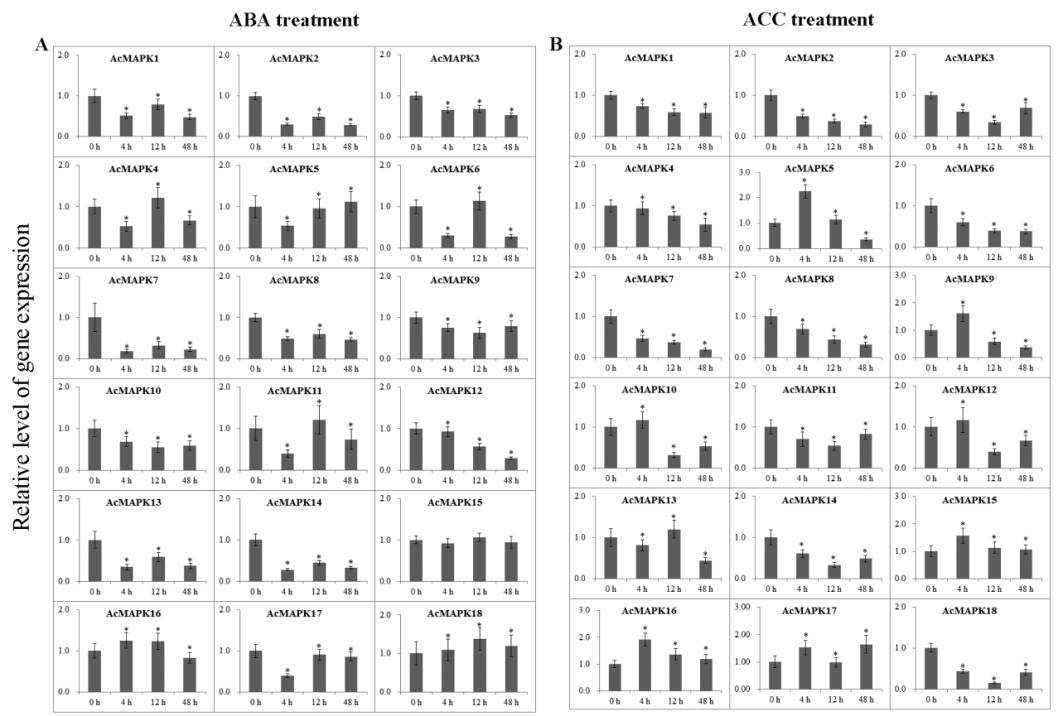


**Figure S4.** Expression profiles of *AcMAPK* genes after ABA and ACC treatment. ABA: treatments with ABA for 4, 12, and 48 h; ACC: treatments with ACC for 4, 12, and 48 h. To visualize the relative expression levels data, 0 h at each treatment was normalized as “1”, and data are means ± SD calculated from three biological replicates. * indicate significant differences in comparison with the control at *p* < 0.05.


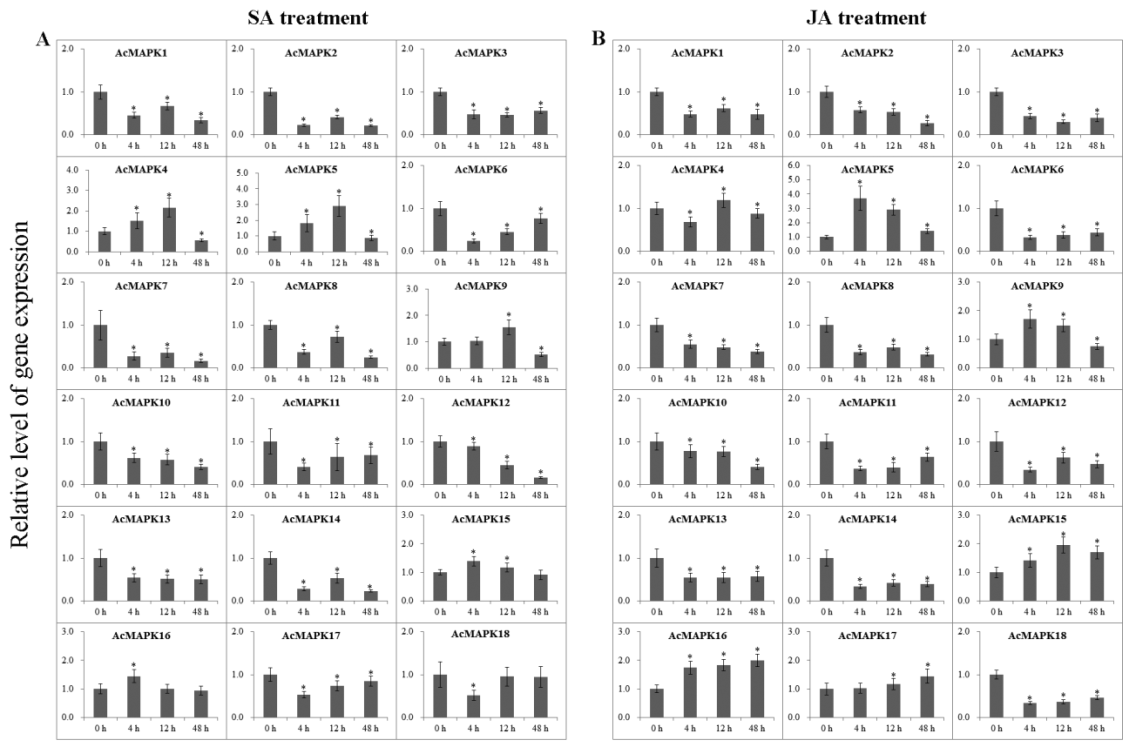


**Figure S5.** Expression profiles of *AcMAPK* genes after SA and JA treatment. SA: treatments with SA for 4, 12, and 48 h; JA: treatments with JA for 4, 12, and 48 h. To visualize the relative expression levels data, 0 h at each treatment was normalized as “1”, and data are means ± SD calculated from three biological replicates. * indicate significant differences in comparison with the control at *p* < 0.05.


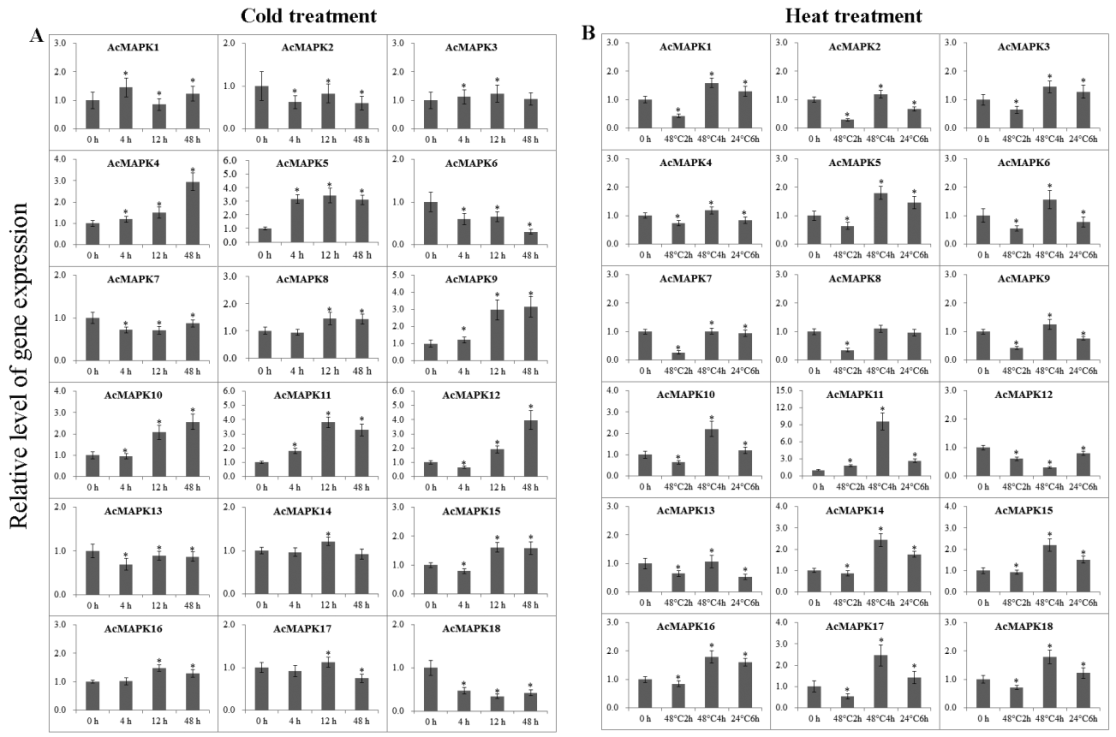


**Figure S6.** Expression profiles of *AcMAPK* genes after Cold and Heat treatment. Cold: treatments with 4 °C for 4, 12, and 48 h; Heat: treatments with at 48 °C for 2 and 4 h, and then at 24°C for another 6 h. To visualize the relative expression levels data, 0 h at each treatment was normalized as “1”, and data are means ± SD calculated from three biological replicates. * indicate significant differences in comparison with the control at *p* < 0.05.


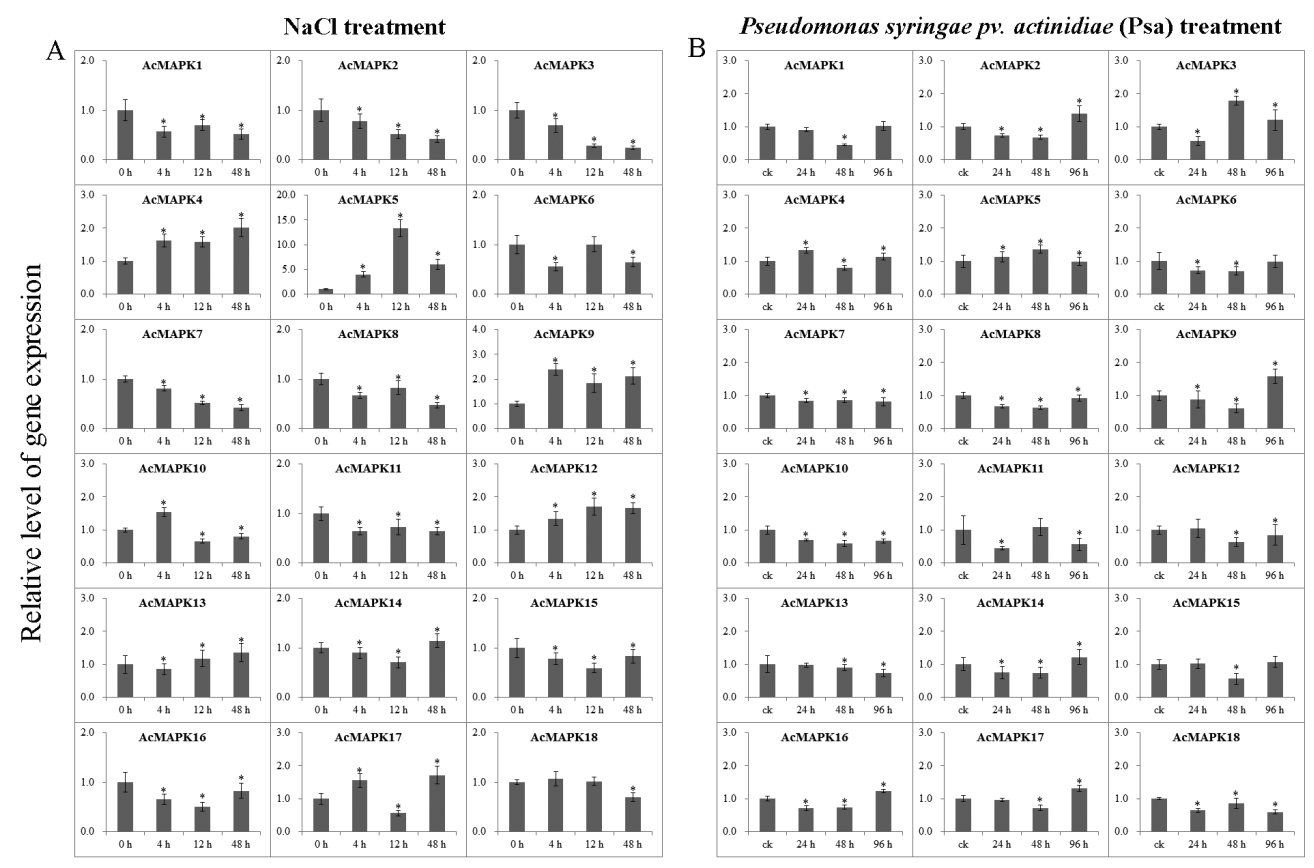


**Figure S7.** Expression profiles of *AcMAPK* genes after salt and Psa treatment. Salt: treatments with NaCl for 4, 12, and 48 h, Psa: *pseudomonas syringae* pv. *actinidiae* infection for 24, 48, and 96 h. To visualize the relative expression levels data, 0 h at each treatment was normalized as “1”, and data are means ± SD calculated from three biological replicates. * indicate significant differences in comparison with the control at *p* < 0.05.
